# Native architecture and acclimation of photosynthetic membranes in a fast-growing cyanobacterium

**DOI:** 10.1093/plphys/kiac372

**Published:** 2022-08-10

**Authors:** Long-Sheng Zhao, Chun-Yang Li, Xiu-Lan Chen, Qiang Wang, Yu-Zhong Zhang, Lu-Ning Liu

**Affiliations:** State Key Laboratory of Microbial Technology, and Marine Biotechnology Research Center, Shandong University, Qingdao 266237, China; Institute of Systems, Molecular and Integrative Biology, University of Liverpool, Liverpool L69 7ZB, UK; Laboratory for Marine Biology and Biotechnology, Pilot National Laboratory for Marine Science and Technology, Qingdao 266237, China; Laboratory for Marine Biology and Biotechnology, Pilot National Laboratory for Marine Science and Technology, Qingdao 266237, China; College of Marine Life Sciences, Ocean University of China, Qingdao 266003, China; State Key Laboratory of Microbial Technology, and Marine Biotechnology Research Center, Shandong University, Qingdao 266237, China; Laboratory for Marine Biology and Biotechnology, Pilot National Laboratory for Marine Science and Technology, Qingdao 266237, China; State Key Laboratory of Crop Stress Adaptation and Improvement, School of Life Sciences, Henan University, Kaifeng 475004, China; Academy for Advanced Interdisciplinary Studies, Henan University, 475004 Kaifeng, China; State Key Laboratory of Microbial Technology, and Marine Biotechnology Research Center, Shandong University, Qingdao 266237, China; Laboratory for Marine Biology and Biotechnology, Pilot National Laboratory for Marine Science and Technology, Qingdao 266237, China; College of Marine Life Sciences, Ocean University of China, Qingdao 266003, China; Institute of Systems, Molecular and Integrative Biology, University of Liverpool, Liverpool L69 7ZB, UK; College of Marine Life Sciences, Ocean University of China, Qingdao 266003, China

## Abstract

Efficient solar energy conversion is ensured by the organization, physical association, and physiological coordination of various protein complexes in photosynthetic membranes. Here, we visualize the native architecture and interactions of photosynthetic complexes within the thylakoid membranes from a fast-growing cyanobacterium *Synechococcus elongatus* UTEX 2973 (Syn2973) using high-resolution atomic force microscopy. In the Syn2973 thylakoid membranes, both photosystem I (PSI)-enriched domains and crystalline photosystem II (PSII) dimer arrays were observed, providing favorable membrane environments for photosynthetic electron transport. The high light (HL)-adapted thylakoid membranes accommodated a large amount of PSI complexes, without the incorporation of iron-stress-induced protein A (IsiA) assemblies and formation of IsiA–PSI supercomplexes. In the iron deficiency (Fe^−^)-treated thylakoid membranes, in contrast, IsiA proteins densely associated with PSI, forming the IsiA–PSI supercomplexes with varying assembly structures. Moreover, type-I NADH dehydrogenase-like complexes (NDH-1) were upregulated under the HL and Fe^−^ conditions and established close association with PSI complexes to facilitate cyclic electron transport. Our study provides insight into the structural heterogeneity and plasticity of the photosynthetic apparatus in the context of their native membranes in Syn2973 under environmental stress. Advanced understanding of the photosynthetic membrane organization and adaptation will provide a framework for uncovering the molecular mechanisms of efficient light harvesting and energy conversion.

## Teaser

Microscopy visualization of the native protein organization and adaptation of the photosynthetic machinery from a fast-growing cyanobacterium reveals its structural heterogeneity and plasticity.

## Introduction

In plants, algae, and cyanobacteria, efficient light energy conversion and rapid electron transport rely on the lateral organization and interactions of photosynthetic macromolecular complexes in the thylakoid membrane, including photosystem I (PSI), photosystem II (PSII), cytochrome *b_6_f* (Cyt *b_6_f*), ATP synthase (ATPase), and type-I NADH dehydrogenase-like complex (NDH-1) (Liu, 2016; Mullineaux and Liu, 2020). Among these photoautotrophs, cyanobacteria show unique advantages in higher efficiency of energy conversion, faster growth, enhanced biomass production, and genetic tractability (Knoot et al., 2018). Given the global energy crisis and climate change, there is an increasing interest in renewable energy and fossil fuel replacement; cyanobacteria have been rapidly developed as a sustainable chassis for producing valuable chemicals and biofuels (Jodlbauer et al., 2021).

*Synechococcus elongatus* UTEX 2973 (Syn2973) has been recently identified as a fast-growing cyanobacterium (Ungerer et al., 2018a,b). Although the genomic sequences of Syn2973 and the model cyanobacterium *S. elongatus* PCC 7942 (Syn7942) are mostly identical except for differences in only 55 genetic loci (Yu et al., 2015), Syn2973 exhibits a three times higher growth rate and more than two-fold higher photosynthetic rate compared with Syn7942 (Ungerer et al., 2018a, 2018b). Moreover, Syn2973 is tolerant of high light (HL), whereas Syn7942 could be severely photoinhibited under HL (Ungerer et al., 2018a).

Studies on the photosynthetic mechanisms of Syn2973 have been performed from the perspective of genetics, physiology, and biochemistry (Yu et al., 2015; Wendt et al., 2016; Mueller et al., 2017; Ungerer et al., 2018a, 2018b). However, the lateral organization of membrane complexes in the Syn2973 thylakoid membranes and the structural plasticity of the photosynthetic machinery, which provide the structural basis for efficient energy conversion and photosynthetic adaptation, remain poorly understood.

Atomic force microscopy (AFM) has unique advantages in probing the native structures of biological membranes and multi-protein complex assemblies as well as their molecular forces and nanomechanics under physiological conditions (Liu et al., 2011; Liu and Scheuring, 2013; Faulkner et al., 2017; Miller et al., 2020). Previous AFM studies have unravelled the structural landscape of thylakoid membranes from Syn7942, *Thermosynechococcus elongatus*, *Synechococcus* sp. PCC 7002, *Synechocystis* sp. PCC 6803, *Prochlorococcus marinus* MED4, and cyanobacterial species capable of far-red light-induced photoacclimation (Casella et al., 2017; MacGregor-Chatwin et al., 2017, 2019, 2022; Ho et al., 2020; Zhao et al., 2020). Here, we report the native arrangements and interactions of electron transport complexes in the thylakoid membranes from Syn2973 visualized by high-resolution AFM. We also systematically study the organizational variability of photosynthetic supercomplex assemblies from low-light (LL)-adapted, HL-adapted, and iron-deficiency-treated Syn2973 cells. Our results provide insight into the construction principles and inter-complex associations that drive the assembly and dynamics of photosynthetic apparatus in Syn2973. Advanced understanding of efficient photosynthesis and environmental adaptation in cyanobacteria would aid in rational design and rewiring of artificial photosynthetic systems to improve photosynthesis and bioenergy production.

## Results

### Architectures of thylakoid membranes from the LL- and HL-adapted Syn2973 cells

IsiA is a membrane-spanning antenna protein associated with PSI in cyanobacteria, forming an IsiA–PSI supercomplex (Toporik et al., 2019; Cao et al., 2020; Jia et al., 2021) to increase optical absorption cross-section (Zhao et al., 2020). The *isiA* gene is widely distributed among cyanobacteria (Chen et al., 2018), and is predominantly expressed under stress conditions (Vinnemeier et al., 1998; Bibby et al., 2001; Boekema et al., 2001; Yousef et al., 2003; Li et al., 2004; Havaux et al., 2005). Syn2973 and Syn7942 have the same genetic organization of the *isiA* gene. The HL regulatory (HLR) sequence and Fur (Ferric Uptake Regulator) box region are located upstream of the *isiA* gene (Supplemental Figure S1), suggesting that iron availability and light intensity play important roles in regulating the expression of *isiA*. Our previous study has confirmed that in the HL or iron deficiency stressed Syn7942 cells, IsiA was highly expressed and formed IsiA–PSI supercomplex assemblies in thylakoid membranes (Zhao et al., 2020).

To study the native architecture and structural adaption of photosynthetic apparatus, thylakoid membranes were isolated from the Syn2973 cells that grew at exponential phase under LL (15 µmol photons m^−2^ s^−1^, 30°C), HL (800 µmol photons m^−2^ s^−1^, 30°C), iron repletion (Fe^+^, 0.31 µmol L^−1^, 40 µmol photons m^−2^ s^−1^, 30°C), and iron deficiency (Fe^−^, 0 µmol L^−1^, 40 µmol photons m^−2^ s^−1^, 30°C) conditions, without any detergent treatment as described previously (Casella et al., 2017; Zhao et al., 2020). We chose the same culturing conditions (i.e. 30°C, air bubbling, and 40 µmol photons m^−2^ s^−1^ for iron-stress conditions) as used for Syn7942 culturing (Zhao et al., 2020), which allowed us to compare the thylakoid membrane architectures and protein content of Syn2973 and Syn7942.

The absorption spectra of Fe^−^-adapted cells exhibited a blue shift of Chl *a* absorption peak (Supplemental Figure S1, B and C), a spectral signature of IsiA expression (Oquist, 1971; Burnap et al., 1993). In contrast, such a blue shift was not observed under HL. Blue native–polyacrylamide gel electrophoresis (BN–PAGE) of detergent-solubilized thylakoid membranes and immunoblot analysis confirmed the presence of IsiA in the Fe^−^ thylakoid membranes but not in HL-adapted thylakoid membranes (Supplemental Figure S1D). These results reveal that in Syn2973 IsiA expression could be induced by Fe^−^, consistent with the finding in Syn7942 and *Synechocystis* sp. PCC 6803 (Ma et al., 2017), whereas IsiA expression in Syn2973 could not be strongly induced under HL, distinct from the finding in Syn7942 (Zhao et al., 2020). Furthermore, the expressed IsiA assemblies associate with PSI to form IsiA–PSI supercomplexes in the Fe^−^ thylakoid membranes; the content of PSI trimers relative to the total protein content in thylakoid membranes was declined under both HL and Fe^−^ conditions, compared to that under LL and Fe^+^ conditions, respectively (Supplemental Figure S1D).

**Figure 1:**
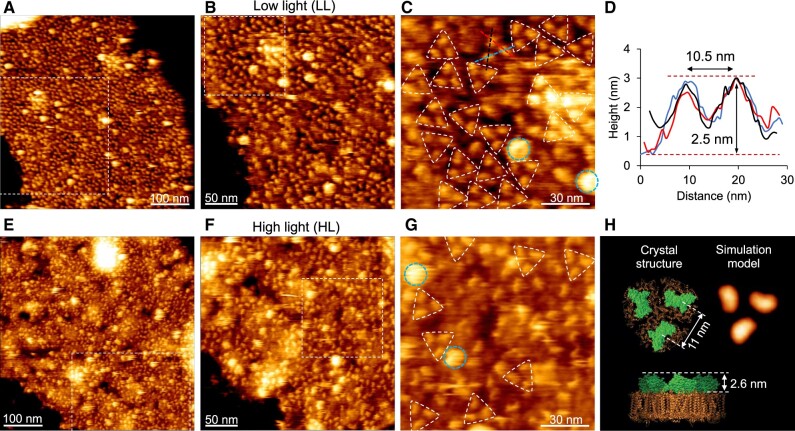
Atomic force microscopy (AFM) topographs of native thylakoid membranes from the LL- and HL-adapted Syn2973 cells. A, AFM topograph showing the densely packed photosynthetic proteins in the LL-adapted thylakoid membrane fragment in liquid. The area indicated by the white box is shown in B. B, High-resolution AFM image of the area highlighted in A, showing the cytoplasmic surface of thylakoid membrane with a crowded arrangement of photosynthetic membrane proteins. The area indicated by the white box is shown in C. C, Zoom-in view of the area highlighted in B, showing the trimeric PSI complexes (triangles) with different orientations. Dashed lines show the positions of height profiles in D. The strongly protruded structures speculated to be the hydrophilic arms of NDH-1 are indicated with blue circles. D, Height profiles corresponding to the dashed lines in C. The lateral distance between peaks of PSI is 10.3 ± 0.8 nm (*n* = 30) and the height of protrusions from the membrane surface is 2.6 ± 0.1 nm (*n* = 30). E, AFM topograph of the cytoplasmic surface of the HL-adapted thylakoid membrane fragment in liquid showing the distribution of photosynthetic proteins. The area indicated by the white box is shown in F. F, High-resolution AFM image of the area highlighted in E, showing the arrangement of photosynthetic membrane proteins. The area delineated by the white box is shown in G. G, Zoom-in view of the area highlighted in F, showing the trimeric PSI complexes (triangles). The strongly protruded structures speculated to be the hydrophilic arms of NDH-1 are indicated with blue circles. H, Atomic structure of the trimeric PSI complex from the cytoplasmic surface (left, PDB: 1JB0). The distance between the highest positions of PSI monomers is 11 nm. The PsaC, PsaD, PsaE subunits are the domains protruding above the membrane surface with a height of 2.6 nm. Simulated AFM image of trimeric PSI complex based on PDB: 1JB0 (right). The representative AFM images were shown from at least five independent membrane preparations.

To understand how photosynthetic complexes are organized in the Syn2973 thylakoid membranes, thylakoid membranes were isolated from both LL- and HL-adapted Syn2973 cells, and were then probed using AFM in liquid (Figure 1). High-resolution AFM imaging enables the determination of not only thylakoid membrane landscape architecture but also the structures and orientations of individual proteins (Supplemental Figure S2) (Zhao et al., 2020). AFM topographs showed that membrane proteins were densely packed in the LL-adapted thylakoid membranes (Figure 1, A and B). PSI is the dominant photosystem in cyanobacteria, and PSI trimers were predominantly observed in the LL-adapted thylakoid membranes (Figure 1C, triangles), with a lateral distance of 10.3 ± 0.8 nm (*n *=* *30) between two protrusions within the trimer and a protruding height of 2.6 ± 0.1 nm (*n *=* *30) above the cytoplasmic membrane surface (Figure 1, D and H) (Jordan et al., 2001). Different orientations and arrangement patterns of PSI trimers were discerned in the LL-adapted thylakoid membranes (Figure 1C;Supplemental Figure S3), resembling the organization of PSI trimers in Syn7942 thylakoid membranes (Zhao et al., 2020).

PSI complexes were less densely packed in the HL-adapted thylakoid membranes than in the LL-adapted thylakoid membranes, and were also randomly distributed in membranes (Figure 1, E and F). Particle counting based on the AFM topographs revealed ∼47% reduction in the PSI trimer content in the HL-adapted membranes than in the LL-adapted membranes (Supplemental Figure S4). Additionally, the content of PSI trimers in HL-adapted Syn2973 thylakoid membranes is higher than that in HL-adapted Syn7942 thylakoid membranes (Zhao et al., 2020). This change is reconciled with the previous finding that the increased photosynthetic electron transport rate in Syn2973 relative to that in Syn7942 was due to the higher content of PSI, Cyt *b_6_f*, and plastocyanin (Ungerer et al., 2018a). No IsiA assemblies were visualized in the Syn2973 HL-adapted membranes, consistent with the spectral and immunoblot results (Supplemental Figure S1).

In addition to the PSI complexes, the structures with the protruding height of 6.0 ± 0.9 nm (*n *=* *40) above the membrane surface were tentatively assigned to be the hydrophobic arms of NDH-1 complexes (Figure 1, C and G, blue circles) (Laughlin et al., 2019; Schuller et al., 2019, 2020; Pan et al., 2020; Zhang et al., 2020; Zhao et al., 2020). The NDH-1 content of HL-adapted thylakoid membranes from Syn2973 was about three-fold higher than that of LL-adapted thylakoid membranes (Supplemental Figure S4M), consistent with previous observations that HL could upregulate the expression of NDH-1 (Hihara et al., 2001; Mi et al., 2001; Liu et al., 2012).

### Arrangement of PSII arrays in thylakoid membranes

**Figure 2:**
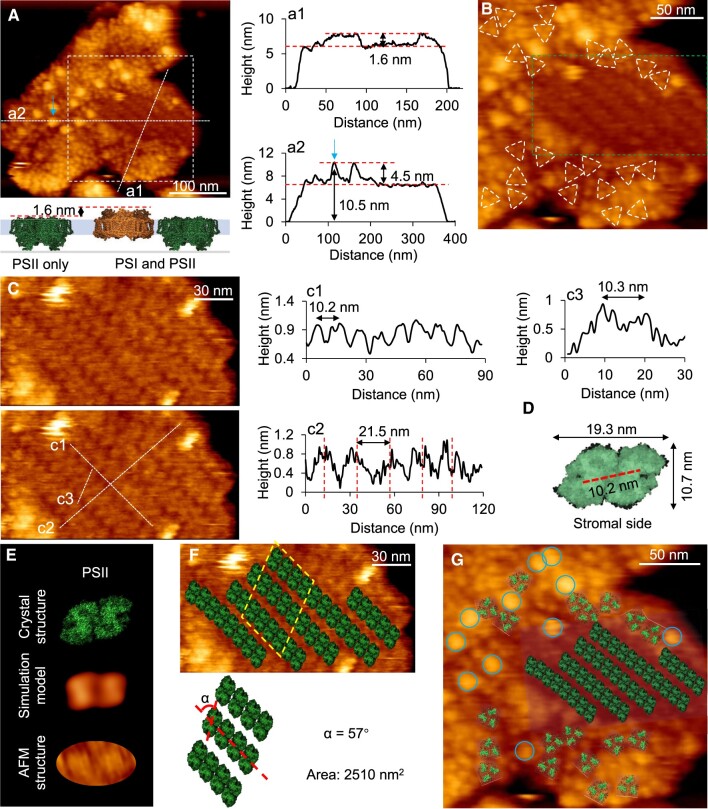
AFM images revealing PSII arrays on the cytoplasmic surface of thylakoid membranes from LL-adapted Syn2973 cells. A, AFM topograph of the cytoplasmic surface of thylakoid membrane fragment showing the densely packed photosynthetic membrane proteins. Height profiles a1, a2 correspond to the dashed lines a1, a2. The heights of putative NDH-1 (blue arrow) from the substrate surface and PSII array surface are 10.5 ± 0.3 nm and 4.5 ± 0.3 nm (*n* = 13), respectively. The height difference between the PSI region and PSII region is 1.6 nm. The area represented by the white box is shown in B. The structural model showing the height difference between the PSII region and PSI–PSII-mixed region when the cytoplasmic surface of thylakoid membrane was exposed to the AFM probe (PSI PDB: 1JB0; PSII PDB: 3WU2). B, Zoom-in view of the area highlighted in A. PSI trimers are highlighted with white triangles. The area indicated by the green box is shown in C. C, AFM image of the area highlighted in B showing the ordered array of PSII dimers. Height profiles c1, c2, c3 correspond to the dashed lines c1, c2, c3. The average distance between adjacent PSII in PSII arrays is 10.2 nm (*n* = 7). The average distance between adjacent PSII arrays is 21.5 nm. The lateral distance between peaks of PSII is 10.4 ± 0.9 nm (*n* = 20). D, Atomic structure of the dimeric PSII complex from the cytoplasmic surface (PDB: 3WU2) with the size shown. The distance between the protrusions of PSII atomic structure is 10.2 nm. E, Atomic structure (top, PDB: 3WU2), simulated AFM images based on PDB structure (middle) and AFM topograph (bottom) of PSII from the cytoplasmic surface. F, Structural model of the arrangement of PSII dimers in regular arrays within the thylakoid membrane. The angle of the extension of PSII arrays and the direction of PSII dimer long axis is 57°. The area occupied by 12 PSII dimers highlighted by the yellow box is 2510 nm^2^. G, Structural model of the arrangement of PSII arrays and PSI trimers within the thylakoid membrane. The putative NDH-1 are indicated by circles.

AFM topographs of the cytoplasmic surface of thylakoid membranes show that some membrane regions are packed with parallel arrays of PSII dimers (Figure 2). The peak-to-peak distance between the two protrusions of the dimeric structure is 10.4 ± 0.9 nm (*n *=* *20), in line with the dimension of PSII dimers (Figure 2, C–E) (Umena et al., 2011). These regions were 1.6 nm on average lower than the surrounding PSI-enriched regions, consistent with the height difference between PSI and PSII at the cytoplasmic surface of thylakoid membranes (Figure 2A). The average center-to-center distance between two coupled PSII dimers within a row is 10.2 nm, and the average interval space between adjacent PSII arrays is 21.5 nm (Figure 2C), in agreement with the organization of PSII arrays observed in Syn7942 (Zhao et al., 2020). The surface area occupied by 12 PSII dimers in the adjacent arrays is ∼2,510 nm^2^ (Figure 2F, yellow diamond), and the angle between the extension of PSII arrays and the direction of PSII dimer long axis is 57° (Figure 2F). This specific tilted angle in a PSII array could be physiologically crucial for the association of light-harvesting antenna supercomplexes—phycobilisomes—with PSII arrays on the cytoplasmic surface (Chang et al., 2015; Zlenko et al., 2017).

Around the PSII arrays and interspersed with PSI assemblies, some highly protruding structures, with the protrusion of 10.5 ± 0.3 nm (*n *=* *13) above the mica surface and 4.5 ± 0.3 nm (*n *=* *13) above the PSII membrane region, were visualized on the cytoplasmic surface of thylakoid membranes (Figure 2A, blue arrow). They were tentatively assigned to be the NDH-1 complexes based on the measured features (Laughlin et al., 2019; Schuller et al., 2019, 2020; Pan et al., 2020; Zhang et al., 2020; Zhao et al., 2020). Such specific thylakoid membrane organization, comprising PSII arrays surrounded by PSI and NDH-1 complexes (Figure 2G), presumably represents the functional photosynthetic assembly unit. Based on the high-resolution AFM topographs, we constructed a structural model of the PSI–PSII–NDH-1 assembly and the association of phycobilisomes with PSII in molecular detail (Supplemental Figure S5). PSI, PSII, and NDH-1 complexes can form direct contacts with each other, whereas phycobilisomes associate with both PSII and PSI to ensure energy flux from phycobilisomes to PSII and PSI. A similar orientation of the PSII–phycobilisome association has been recently recorded in cryo-electron tomography of the phycobilisome–PSII supercomplex from red algae (Li et al., 2021).

**Figure 3:**
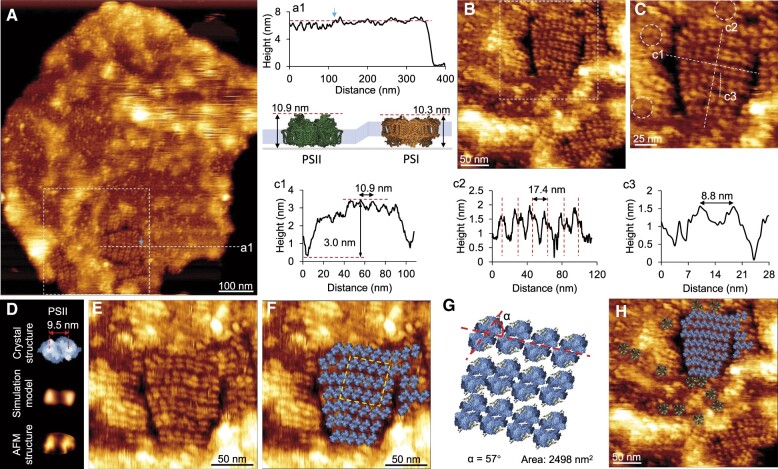
AFM images revealing PSII arrays on the lumenal surface of thylakoid membranes from LL-adapted Syn2973 cells. A, AFM topograph of the thylakoid membrane lumenal surface showing the structures with an ordered arrangement. Height profile a1 corresponds to the dashed line a1. Blue arrow indicates the interface of ordered arrays and disordered regions. The area represented by the white box is shown in B. The structural model shows the heights of PSII region and PSI region when the cytoplasmic surface of thylakoid membrane sticks to the substrate (PSI PDB: 1JB0; PSII PDB: 3WU2). B, AFM topograph of the area highlighted in A showing the ordered arrays of PSII dimers. The area represented by the white box is shown in C. C, Zoom-in view of the area highlighted in B, showing the ordered arrays of PSII dimers. PSI trimers are highlighted with white circle. Height profiles c1, c2, c3 correspond to the dashed lines c1, c2, c3. The average distance between adjacent PSII in PSII arrays is 10.9 nm. The height of dimers from the membrane surface is 3.0 ± 0.1 nm (*n* = 5). The average distance between adjacent PSII arrays is 17.4 nm. The lateral distance between peaks of PSII is 8.5 ± 0.5 nm (*n* = 20). D, Atomic structure (top, PDB: 3WU2), simulated AFM image based on PDB (middle) (Zhao et al., 2020) and AFM topograph (bottom) of PSII from the lumenal surface. The distance of protrusions in the PSII crystal structure is shown. E, High-resolution AFM image of PSII dimer arrays. Same membrane area as C, but the scan angle is set to 90°. F, Structural model of the arrangement of PSII dimers in ordered arrays within the thylakoid membrane (PDB: 3WU2). The area represented by yellow box is shown in G. G, Structural model of the arrangement of PSII arrays highlighted in F. The angle of the extension of PSII arrays and the direction of PSII dimer long axis is 57°. The area occupied by 12 PSII dimers highlighted by the yellow box is 2,498 nm^2^. H, The arrangement of PSII arrays and PSI trimers within the thylakoid membrane.

The long-range arrays of PSII dimers were also imaged by AFM scanning on the lumenal surface of thylakoid membranes (Figure 3), and the organization of PSII arrays was not affected by the direction of AFM scanning (Figure 3, A, B and E). The height of the membrane region accommodating PSII arrays is comparable with that of the PSI-enriched membrane region (Figure 3A). The distance between the two protrusions of the dimeric structure is 8.5 ± 0.5 nm (*n *=* *20), a typical dimension of PSII dimers (Figure 3, C and D). The average distance between adjacent dimers in the PSII array is 10.9 nm, and the height of dimers above the membrane surface is 3.0 ± 0.1 nm (*n *=* *5) (Figure 3C). The average distance between adjacent PSII arrays is 17.4 nm (Figure 3C), less than that observed on the cytoplasmic surface. The angle between the extension of PSII arrays and the direction of PSII dimer long axis is 57° (Figure 3, F and G, Supplemental Figure S6); the surface area occupied by 12 PSII dimers in PSII arrays observed from the lumenal surface is ∼2,498 nm^2^ (Figure 3, F and G, yellow diamond); these features are comparable to that observed on the cytoplasmic side (Figure 2F). In addition, PSI trimers are distributed around the PSII arrays, and some PSI complexes form direct contacts with PSII structures, revealing their inter-complex association in the context of native membranes (Figure 3H;Supplemental Figure S5, C and D).

### Aberrant membrane orientation of PSI complexes

**Figure 4:**
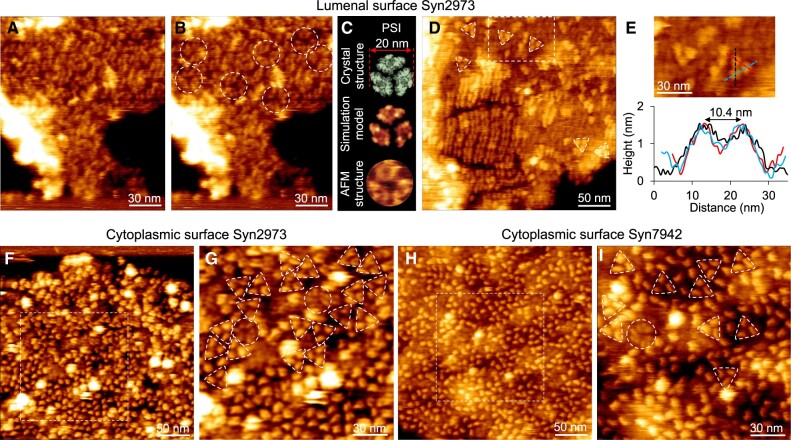
AFM images reveal the “upside-down” PSI in thylakoid membranes. A, High-resolution AFM image of the lumenal surface of thylakoid membranes from HL-adapted Syn2973. B, The same image as A with PSI trimers highlighted by circles. C, Atomic structure (top, PDB: 1JB0), simulated structure based on PDB (middle) (Zhao et al., 2020) and AFM topograph (bottom) of PSI from the lumenal surface. The lateral size of PSI crystal structure is shown. D, AFM topograph of the lumenal surface of the LL-adapted thylakoid membrane. This panel is the zoom-in view of Figure 3A, and shows the same membrane area as depicted in Figure 3, B, C, and E. Trimeric PSI structures with the cytoplasmic side facing to the AFM probe are highlighted by triangles. The area represented by the white box is shown in E. E, Zoom-in view of the area highlighted in D showing trimeric PSI complexes (triangles). Dashed lines show the positions of height profiles. The lateral distance between peaks of PSI is 10.4 nm. F, High-resolution AFM image of the cytoplasmic surface of thylakoid membrane from LL-adapted Syn2973 showing the densely packed photosynthetic membrane proteins. Area represented by the white box is shown in G. G, Zoom-in view of the area highlighted in F, showing the photosynthetic complexes in detail. Trimeric PSI structures with the cytoplasmic side facing the AFM probe are highlighted by triangles. Trimeric PSI structures with the lumenal side exposing to the AFM probe are highlighted by circles. H, High-resolution AFM image of the cytoplasmic surface of thylakoid membranes from LL-adapted Syn7942 showing the densely packed photosynthetic membrane proteins. Area represented by the white box is shown in I. I, Zoom-in view of the area highlighted in H showing the membrane organization of photosynthetic complexes in detail. Trimeric PSI structures with the lumenal side facing the AFM probe are highlighted by triangles. Trimeric PSI structures with the lumenal side facing the AFM probe are highlighted by circles. The representative AFM images were shown from at least five independent membrane preparations.

AFM imaging on the lumenal surface of thylakoid membranes revealed two groups of dimeric protrusions with the peak-to-peak distance of protrusions of 9.9 ± 0.7 nm (*n *=* *30) (blue oval) and 6.4 ± 1.2 nm (*n *=* *12) (white oval), assigned as PSII dimers and Cyt *b_6_f* dimers, respectively (Supplemental Figures S7 and S8). In addition, based on the high-resolution AFM imaging, we could differentiate the cytoplasmic and lumenal structures of PSI complexes (Supplemental Figure S2). Closer inspection showed that some PSI trimers have the opposite membrane-spanning orientation as most PSI trimers in native thylakoid membranes, with the cytoplasmic side (peak-to-peak distance of three protrusions: 10.4 nm) exposed on the lumenal surface of thylakoid membranes (Figure 4, A–E) or vice versa (Figure 4, F and G; Supplemental Figures S7 and S8). These “upside-down” PSI complexes were interspersed with “normal” PSI, PSII, and Cyt *b_6_f*, and establish close contacts with these photosynthetic complexes (Supplemental Figure S8, C–F), excluding the possibility that the opposing PSI complexes were generated by externally induced membrane reorganization, such as physical fusion of two thylakoid membrane patches with opposite orientations. Consistently, the aberrantly oriented PSI structures have also been identified in the Syn7942 thylakoid membranes (Figure 4, H and I; Supplemental Figure S8, G and H). These results reflect the general features of the flexibility and dynamics of PSI assembly and integration into native thylakoid membranes, which may provide the structural basis for fine-tuning photosynthetic performance and fitness.

### Architectures of thylakoid membranes from the iron-replete and iron-starved Syn2973 cells

**Figure 5:**
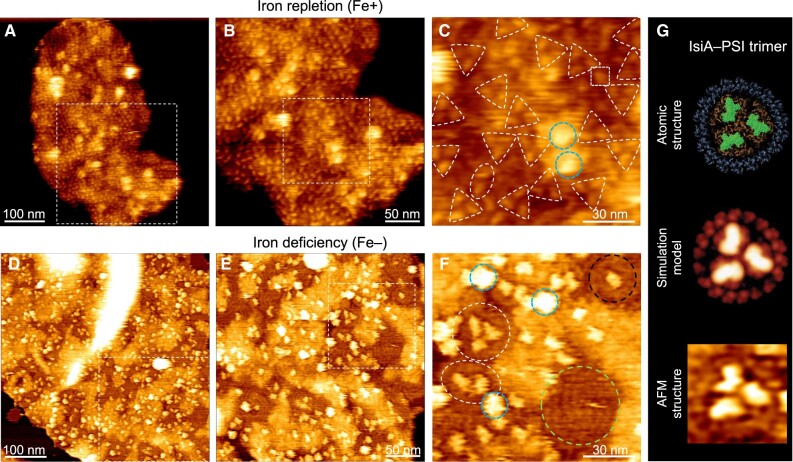
AFM topographs of native thylakoid membranes from the iron-replete (Fe^+^) and iron-starved (Fe^−^) Syn2973 cells. A, AFM topograph of the Fe^+^ thylakoid membrane fragment in liquid. The area indicated by the white box is shown in B. B, High-resolution AFM image of the cytoplasmic surface of the area highlighted in A, showing the crowded arrangement of photosynthetic membrane proteins. The area represented by the white box is shown in C. C, Zoom-in view of the area highlighted in B, showing the trimeric (triangle), dimeric (oval), and monomeric (square) PSI complexes. The strongly protruded structures speculated to be the hydrophilic arms of NDH-1 complexes are highlighted with blue circles. D, AFM topograph of the Fe^−^ thylakoid membrane fragment in liquid. The area indicated by the white box is shown in E. E, High-resolution AFM image of the cytoplasmic surface of the area highlighted in D. The area indicated by the white box is shown in F. F, Zoom-in view of the area highlighted in E, showing an IsiA–PSI trimer (large circle on the left side), an IsiA–PSI dimer (oval) and an IsiA–PSI monomer (large circle in the top right corner). IsiA self-assemblies are highlighted with large circles in the bottom right corner. The strongly protruded structures speculated to be the hydrophilic arms of NDH-1 complexes are highlighted with small circles. G, Structural analysis of the IsiA–PSI supercomplex. Top, atomic structure of IsiA–PSI from the cytoplasmic surface (PDB: 6NWA); middle, simulated AFM image of IsiA–PSI supercomplex (Zhao et al., 2020) based on the PDB structure; bottom, zoom-in view of AFM topograph of an IsiA–PSI supercomplex. The representative AFM images were shown from at least five independent membrane preparations.

Expression of IsiA is crucial for the photosynthetic activities and growth of cyanobacteria in response to various environmental conditions, including iron deficiency, oxidative stress, high salt, heat stress, and HL (Vinnemeier et al., 1998; Park et al., 1999; Li et al., 2004; Havaux et al., 2005; Behrenfeld et al., 2006; Chen et al., 2018; Zhao et al., 2020). Our results showed that the Fe^−^ stress could induce IsiA expression in Syn2973 (Supplemental Figure S1). AFM imaging revealed the dense packing of photosynthetic membrane complexes in both Fe^+^ and Fe^−^ Syn2973 thylakoid membranes (Figure 5; Supplemental Figure S9). The Fe^+^ thylakoid membranes contain predominantly PSI trimers without IsiA assemblies (Figure 5, A–C). In contrast, the PSI trimer content in the Fe^−^ thylakoid membranes decreased by 91% (Supplemental Figure S4, G–M) and IsiA assemblies became dominant in the membrane area enriched with PSI complexes (Figure 5, E and F; Supplemental Figure S4K, 9, A and B), forming the IsiA–PSI supercomplexes as observed in the Fe^−^ and HL-adapted Syn7942 thylakoid membranes (Zhao et al., 2020). A typical IsiA–PSI trimer assembly comprises 18 IsiA proteins surrounding a PSI trimer (Figure 5F, white circle; Figure 5G;Supplemental Figure S9B, white circle) (Toporik et al., 2019). IsiA–PSI dimers, IsiA–PSI monomers, and IsiA-only assemblies were also observed in the Fe^−^ Syn2973 thylakoid membranes, confirming the dynamic association between IsiA and PSI (Figure 5F).

PSI trimers, PSII dimers, and Cyt *b_6_f* dimers were also visualized from the lumenal surface of Fe^−^ thylakoid membrane (Supplemental Figure S9, C–F). PSII dimers and Cyt *b_6_f* dimers (Supplemental Figure S9F, blue ovals) form direct contacts with IsiA proteins. The ring-like structures, tentatively assigned to the F_0_ domain of ATPase, were recorded from both the cytoplasmic and lumenal surfaces of thylakoid membranes (Supplemental Figure S9, blue arrows), due to the transmembrane feature of the ATPase F_0_ domain (Hahn et al., 2018).

Putative NDH-1 complexes, with the protrusion of 6.1 ± 0.6 nm (*n *=* *40) above the membrane, were observed in both Fe^+^ and Fe^–^ thylakoid membranes (Figure 5, C and F, blue circles), and the Fe^–^ thylakoid membranes contain a higher content of NDH-1 complexes than the Fe^+^ thylakoid membranes (Supplemental Figure S4M). High-resolution AFM images show that a highly protruding domain of the NDH-1 complex is accompanied by two small protrusions (Supplemental Figure S10A, oval; 10B), as visualized by AFM imaging of Syn7942 thylakoid membranes (Zhao et al., 2020) and cryo-electron microscopy (cryo-EM) of NDH-1 (Supplemental Figure S10, D and F, PDB: 6HUM) (Schuller et al., 2019). NDH-1 complexes with the CUP domain were often seen in Fe^–^ thylakoid membranes (Supplemental Figure S10C). The height of the carbon uptake (CUP) domain above membrane surface is 4.3 ± 0.4 nm (*n *=* *20), greater than that of the PSI protrusion (2.6 ± 0.1 nm, *n *=* *30) (Supplemental Figure S10C). The distance between the NDH-1 hydrophobic arm and CUP domain is 13.3 ± 0.8 nm (*n *=* *20) (Supplemental Figure S10C), in line with the cryo-EM structure of NDH-1 (Supplemental Figure S10, E and F, PDB: 6TJV) (Schuller et al., 2020). The close association between NDH-1 and IsiA–PSI to form the NDH-1–IsiA–PSI supercomplex provides the structural foundation for efficient cyclic electron transfer (Supplemental Figure S10, G and H). Moreover, various assembly patterns of the NDH-1 and IsiA–PSI supercomplexes were characterized in the thylakoid membranes (Supplemental Figure S11).

## Discussion

The fast-growing cyanobacterium Syn2973 has been considered as a potential candidate for “microbial cell factories” in biotechnological applications. Syn2973 is tolerant of HL and grows three times faster than Syn7942, and its photosynthetic efficiency is more than twofold higher than Syn7942 at optimal growth conditions (Ungerer et al., 2018a, 2018b). To compensate for the severe loss of PSI, in HL-adapted Syn7942 the remaining PSI bind with IsiA forming IsiA–PSI supercomplexes to increase the optical absorption cross-section (Zhao et al., 2020). In contrast, IsiA was not expressed in Syn2973 grown under HL (Figure 1, E–G, Supplemental Figure S1, B–D), and the HL-adapted thylakoid membranes possess increased PSI content compared with HL-adapted Syn7942, as revealed by AFM imaging (Supplemental Figure S4M) and spectroscopy (Ungerer et al., 2018a), suggesting a strategy for improving photosynthetic efficiency and growth of cyanobacterial cells.

The expression of IsiA in cyanobacteria could be induced by accumulation of reactive oxygen species (ROS) in cells grown under HL (Havaux et al., 2005; Wang et al., 2008). The absence of IsiA in HL-adapted Syn2973 may indicate a low level of ROS in HL-adapted Syn2973, resulted from a faster photosynthetic electron transport instead of passing electrons onto oxygen. In addition, the NAD^+^ kinase of Syn2973 exhibits improved kinetics for generating a large pool of NADP^+^ to accept electrons from photosynthetic linear electron flow; Syn2973 has higher ATP-producing activity and an elevated content of NADPH than Syn7942, ultimately facilitating photosynthetic carbon fixation (Ungerer et al., 2018b). Our AFM results also revealed that the NDH-1 content increased markedly in HL-adapted Syn2973 thylakoid membranes (Supplemental Figure S4M), probably resulting in the increase of NDH-1-dependent cyclic electron flux around PSI (Hihara et al., 2001; Mi et al., 2001; Liu et al., 2012; Zhang et al., 2020). This alteration may lead to the consumption of excess electrons at the acceptor side of PSI, thereby preventing accumulation of ROS (Martin et al., 2004; Latifi et al., 2009). In contrast, expression of IsiA proteins in Syn2973 was induced by iron deficiency (Figure 5, D–F), as found in other cyanobacterial species (Burnap et al., 1993; Leonhardt and Straus, 1994; Singh et al., 2003; Chen et al., 2018; Zhao et al., 2020). It has been suggested that iron-starved cyanobacteria also suffer from oxidative stress (Latifi et al., 2005), and redox imbalance may be the broad spectrum inducer of IsiA expression (Havaux et al., 2005). The electrons produced in the linear electron transport chain may not be utilized effectively due to the decreased PSI content in Fe^–^ Syn2973, and the excess electrons may cause accumulation of ROS, resulting in expression of IsiA.

Cyanobacterial thylakoid membranes are structurally heterogeneous and highly dynamic, and are formed in vivo following stepwise biogenesis pathways (Liu, 2016; Mullineaux and Liu, 2020; Huokko et al., 2021; Zabret et al., 2021; Zhang et al., 2021; Rahimzadeh-Karvansara et al., 2022). AFM imaging has delineated diverse assembly patterns and organizational heterogeneity of photosynthetic complexes in thylakoid membranes from different cyanobacterial species, as reflected mainly by the lateral segregation of PSII and PSI. The PSI trimers showed relatively random orientations in the PSI-enriched thylakoid membranes from Syn2973, Syn7942, *Synechocystis* sp. PCC 6803, *Prochlorococcus marinus* MED4, as well as far-red light-acclimated *Chroococcidiopsis thermalis* PCC 7203 and *Chlorogloeopsis fritschii* PCC 9212 (Casella et al., 2017; MacGregor-Chatwin et al., 2017, 2019, 2022; Zhao et al., 2020), in contrast to the more regular arrangement of PSI trimers observed in the thylakoid membranes of *Thermosynechococcus elongatus*, *Synechococcus* sp. PCC 7002, *Prochlorococcus marinus* MIT9313 and SS120, as well as far-red light-acclimated *Synechococcus* 7335 (MacGregor-Chatwin et al., 2017, 2019, 2022). The PSII dimers show both random and crystallized arrangements in the Syn2973 thylakoid membranes (Figures 2 and 3; Supplemental Figures S7 and S8). Given their distinct absorption and turnover rates, such lateral segregation of PSI and PSII may provide special local membrane environments for energy conversation and electron flow (Busch et al., 2013). The large regions of defined PSII arrays observed in AFM (Figures 2 and 3) are consistent with previous AFM studies on grana thylakoids (Sznee et al., 2011), and on thylakoids from *Synechococcus* 7335 (Ho et al., 2020). Such arrays were also observed in a previous EM study of crystalline PSII arrays in *Synechocystis* sp. PCC 6803 (Folea et al., 2008), and they resemble the compartmentalization of PSII in the grana lamellae of plants (Dekker and Boekema, 2005; Kouril et al., 2012; Johnson et al., 2014; Levitan et al., 2019). These regular arrangements of photosystem complexes not only elevate the local PSII content in some thylakoid membrane regions but also provide the framework for docking of multiple phycobilisomes to construct photosynthetic assembly units, as seen in Supplemental Figure S5, B and D and also in Ho et al. (2020). Such units are composed of phycobilisome arrays, PSII dimer arrays, and surrounding PSI complexes to ensure efficient photosynthesis and state transitions locally.

The heterogeneity of cyanobacterial thylakoid membranes was also reflected by the structural variety of PSI–NDH-1 assemblies and mixed membrane-spanning orientations of PSI complexes. In Syn2973 thylakoid membranes, PSI–NDH-1 supercomplexes with various assembly forms were visualized (Supplemental Figures S4, 10, and 11), consistent with the finding on Syn7942 thylakoid membranes (Zhao et al., 2020). The association between PSI and NDH-1 and their flexible, dynamic assembly in native thylakoid membranes could facilitate the NDH-1-dependent cyclic electron transport and physiologically balance the ATP/NADPH ratio required for the Calvin–Benson cycle in response to the changing environments (Peng et al., 2008, 2009; Kouril et al., 2014; Gao et al., 2016; Yadav et al., 2017).

Our study provides evidence for the “upside-down” PSI complexes and variable insertion of photosynthetic complexes into thylakoid membranes. The proper orientation of PSI is essential for electron transfer from plastocyanins in thylakoid lumen to ferredoxins in the cytoplasm (Supplemental Figure S2), and the “upside-down” PSI complexes are unlikely to play the same role as “normal” PSI. Consistently, only a small amount of “upside-down” PSI were observed together with major “normal” PSI complexes in cyanobacterial thylakoid membranes. The mechanisms underlying protein integration into thylakoid membranes and the actual physiological function of “upside-down” PSI complexes merit further investigation.

In conclusion, we performed in-depth AFM imaging to unravel the supramolecular organization and variability of photosynthetic complexes in native thylakoid membranes from the fast-growing cyanobacterium ecotype Syn2973, which underpin efficient photosynthesis in different light and iron availability conditions. PSI trimers are predominant in Syn2973 thylakoid membranes, and lateral segregation of PSI and PSII were mainly observed in addition to inter-complex associations of PSI and PSII. HL-adapted thylakoid membranes have a high content of PSI complexes without IsiA assemblies; Fe^−^ thylakoid membranes contain a low abundance of PSI complexes associated with IsiA proteins, forming IsiA–PSI supercomplexes with various structures. The structural plasticity and dynamics of thylakoid membranes were further indicated by the visualization of diverse PSI–NDH-1 assemblies and aberrant membrane orientation of PSI complexes in thylakoids. Advanced understanding of the architecture and modularity of cyanobacterial thylakoid membranes that conduct efficient photosynthesis is essential for unveiling the molecular mechanisms of photosynthetic electron flow and adaptation, and will inform rational design and construction of artificial photosynthetic systems for sustainable biofuel production.

## Materials and methods

### Strains and growth conditions

*Synechococcus elongatus* UTEX 2973 (Syn2973) and *S. elongatus* PCC 7942 (Syn7942) were grown in BG11 medium at 30°C. The cells were cultivated in a photobioreactor Multi-Cultivator MC 1000-OD (Photon Systems Instruments, Brno, Czech Republic), with continuous air bubbling and constant cool white light at a starting optical density (OD)_750_ = 0.2. LL and HL-adapted Syn2973 cells were grown under 15 µmol photons⋅m^−2^⋅s^−1^ and 800 µmol photons⋅m^−2^⋅s^−1^, respectively. Iron-replete Syn2973 and Syn7942 cells were cultured under 40 µmol photons⋅m^−2^⋅s^−1^ in BG11 with Fe^3+^ concentration of 3.12 µmol L^−1^. Iron-starved Syn2973 cells were cultured under 40 µmol photons⋅m^−2^⋅s^−1^ in iron-free BG11 medium. Iron-free BG11 medium was prepared as previously reported with a slight modification (Katoh et al., 2000). The medium without citric acid, ferric citrate, MgSO_4_, CaCl_2_, K_2_HPO_4_, and trace elements was treated with Chelex 100 resin (Bio-Rad, Hercules, Calif.) and then add MgSO_4_, CaCl_2_ and trace elements. After autoclave, the final iron-free BG11 was ready after the supplementation of K_2_HPO_4_ (treated with Chelex 100 resin, filtration sterilization). Cells were cultured in iron-free medium for iron-deficiency acclimatization (Wang et al., 2010; Zhao et al., 2020). The iron-replete cells at the mid-logarithmic growth phase were washed four times with iron-free BG11 and then grown in iron-free BG11.

### Thylakoid membrane isolation

For the isolation of thylakoid membranes, 40-mL cells at OD_750_ = 1.0 were pelleted, washed with buffer A (50-mM MES-NaOH, pH 6.5, 5-mM CaCl_2_, 10-mM MgCl_2_) twice, and then resuspended in 500-μL buffer A containing 25% glycerol (v/v) and protease inhibitor, followed by cell breakage using glass bead (212–300 μm in diameter, Sigma, G1277, America) at 4°C by vortex eight times at the highest speed for 25 s with 1 min cooling on ice between the runs. The mixture was then centrifuged at 3,000*g* for 2 min to remove the glass beads and unbroken cells. Crude thylakoid membranes were further purified by sucrose-gradient centrifugation (230,500*g*, Beckman RPS40 rotor) at 4°C with a step sucrose gradient (2.0, 1.3, 1.0, and 0.5 M). The Chl-enriched samples at the 1.0–1.3 M sucrose interface were collected and used for subsequent analysis. Detergent was avoided during membrane isolation and AFM imaging to ensure the physiological organization of thylakoid membranes.

### Atomic force microscopy (AFM)

Freshly cleaved mica surface was immersed in 38 μL of adsorption buffer (10-mM Tris–HCl, pH 7.5, 150-mM KCl, 25-mM MgCl_2_) and 2 μL of thylakoid membrane samples were immediately injected into the buffer drop and then incubated for 1.5 h in a humidor at room temperature. After adsorption, the sample was carefully rinsed with 800-μL imaging buffer to remove the free membranes (10-mM Tris–HCl, pH 7.5, 150-mM KCl) (Liu et al., 2009, 2011; Zhao et al., 2016, 2020; Miller et al., 2020). Then, the membranes on mica were imaged using JPK NanoWizard 3 AFM in AC imaging mode in the imaging buffer at room temperature. AFM was equipped with an ULTRA S scanner and Ultra-Short Cantilever probes (0.3 N·m^−1^, Nano World). The tip spring constant was routinely calibrated. High-resolution imaging was performed at a scan rate of 5 Hz and a resolution of 512 × 512 pixels. Images were processed with JPK SPM Data Processing (JPK). The construction of models was carried out by Chimera and Adobe Illustrator. Simulations of AFM images were carried out as previously described (Zhao et al., 2020).

### Absorption spectra

Absorption spectra were recorded at room temperature with 1-nm increment using a Cary UV-Vis Spectrophotometer (Agilent Technologies, USA). The OD_750_ of cells was adjusted to 0.5 before measurement.

### Blue native–PAGE and immunoblot analysis

Thylakoid membrane proteins in their native form were studied by blue native–PAGE according to the previous methods (Zhang et al., 2012) with the exception that 3% (w/v) n-Dodecyl-β-d-maltoside (Anagrade, D310, USA) was used for solubilization (Zhao et al., 2020; Huokko et al., 2021). Precast NativePAGE Bis–Tris protein gels with 4%–16% (w/v) gradient (NativePAGE, Thermo Fisher) were used to separate protein complexes. The voltage was gradually increased from 50 V up to 200 V during the gel run. After electrophoresis, proteins were transferred to a polyvinylidene fluoride (PVDF) membrane (Immobilon-P, Millipore) and analyzed with antibodies specific to IsiA (ImmunoGen Biological Technology) and PsaB (Agrisera, AS10 695, Sweden).

### Accession numbers

Sequence data from this article can be found in the GenBank databases under the following accession numbers: *isiA* (M744_08705).

## Supplemental data

The following materials are available in the online version of this article.

**Supplemental Figure S1.** Syn2973 acclimates to LL, HL, Fe^+^, and Fe^–^ conditions.

**Supplemental Figure S2.** Determination of the cytoplasmic and lumenal surfaces of cyanobacterial thylakoid membranes.

**Supplemental Figure S3.** Arrangement of PSI trimers in thylakoid membranes from LL-adapted Syn2973 cells.

**Supplemental Figure S4.** The organization of PSI trimers and putative NDH-1 in thylakoid membranes from LL, HL, Fe^+^ and Fe^–^-adapted Syn2973 cells.

**Supplemental Figure S5.** Association of PSI and PSII dimer arrays in thylakoid membranes from LL-adapted Syn2973 cells.

**Supplemental Figure S6.** AFM images revealing the PSII arrays on the lumenal surface of thylakoid membranes from LL-adapted Syn2973 cells.

**Supplemental Figure S7.** AFM image of the lumenal surface of thylakoid membrane from HL-adapted Syn2973 cells showing the distribution of photosynthetic membrane proteins.

**Supplemental Figure S8.** AFM images revealing the PSII, cytochrome *b_6_f* (Cyt *b_6_f*) and upside-down PSI complexes in thylakoid membranes.

**Supplemental Figure S9.** AFM images of thylakoid membranes from iron-starved (Fe^–^) Syn2973 cells.

**Supplemental Figure S10.** AFM images of putative NDH-1 in Fe^–^ thylakoid membranes from Syn2973 cells.

**Supplemental Figure S11.** Variability of the association of PSI and NDH-1 in thylakoid membranes from iron-starved Syn2973 cells.

## Supplementary Material

kiac372_Supplementary_Data

## Notes

L.-S.Z., Y.-Z.Z. and L.-N.L. conceived the project; L.-S.Z. C.-Y.L. and L.-N.L. performed the research; L.-S.Z., Q.W., X.-L.C. and L.-N.L. analyzed the data; L.-S.Z., Y.-Z.Z. and L.-N.L. wrote the manuscript.

The author responsible for distribution of materials integral to the findings presented in this article in accordance with the policy described in the Instructions for Authors (https://academic.oup.com/plphys/pages/general-instructions) is Lu-Ning Liu.
