# Supplementary material for: Native architecture and acclimation of photosynthetic membranes in a fast-growing cyanobacterium
Source: Plant Physiol. 2022 Aug 10;190(3):1883–95. doi: 10.1093/plphys/kiac372 (PMC9614513; doi:10.1093/plphys/kiac372)
Supplement: kiac372_Supplementary_Data [file kiac372_supplementary_data.pdf]

Supplemental Data

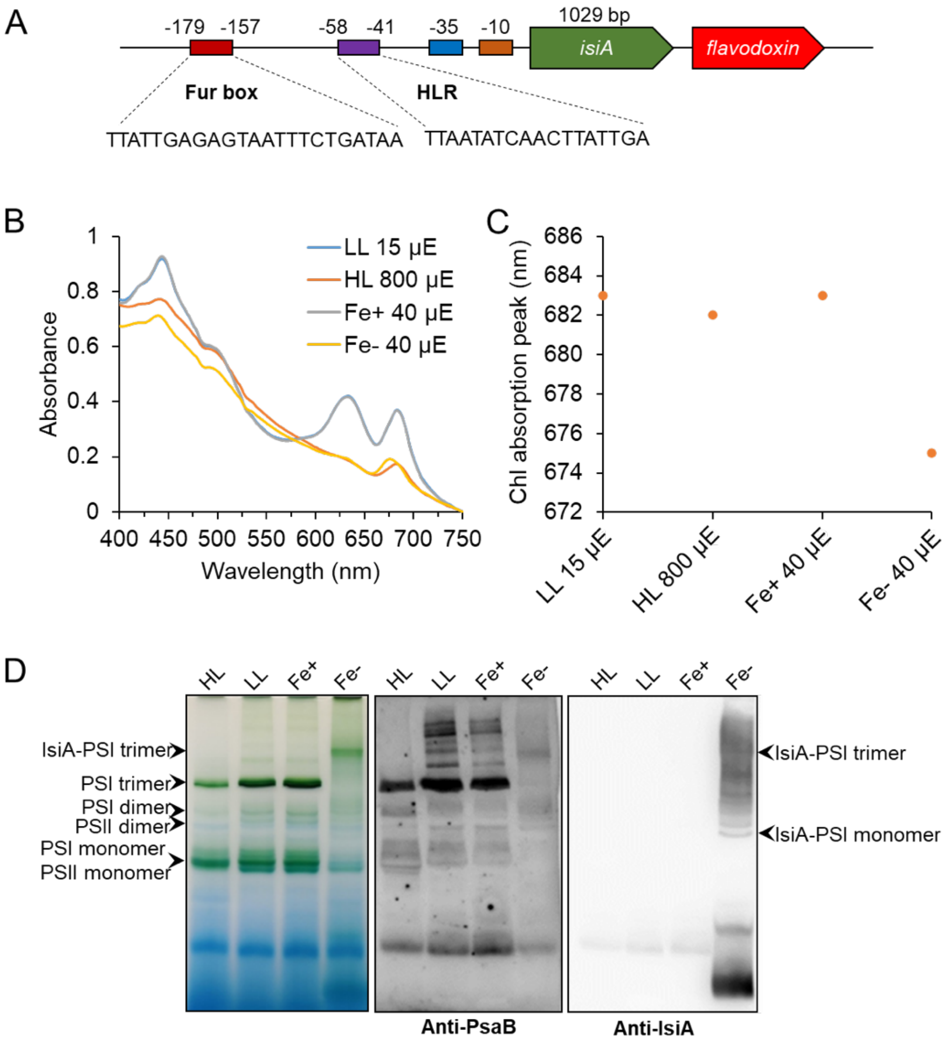

Supplemental Figure S1. Syn2973 acclimates to low light (LL), high light (HL), Fe+ and Fe– conditions. A, Genetic organization of the *isiA* gene (*M744_08705*). The high-light regulatory (HLR) sequence and Fur (Ferric Uptake Regulator) box region are located upstream of the *isiA* gene. **B**, Room-temperature absorption spectra of LL, HL, Fe+ and Fe–-adapted cells. The cell concentration was unified to the same OD_{750} (0.5). **C**, Absorption peaks of Chl *a* at 680 nm achieved from the absorption spectra shown in **B**. The representative results in **B** and **C** were shown from three biological replicates. **D**, BN-PAGE of photosynthetic complexes from DM-solubilized thylakoid membranes and immunoblot analysis using the antibodies specific to PsaB and IsiA. 75 μg solubilized membrane proteins were loaded per sample.

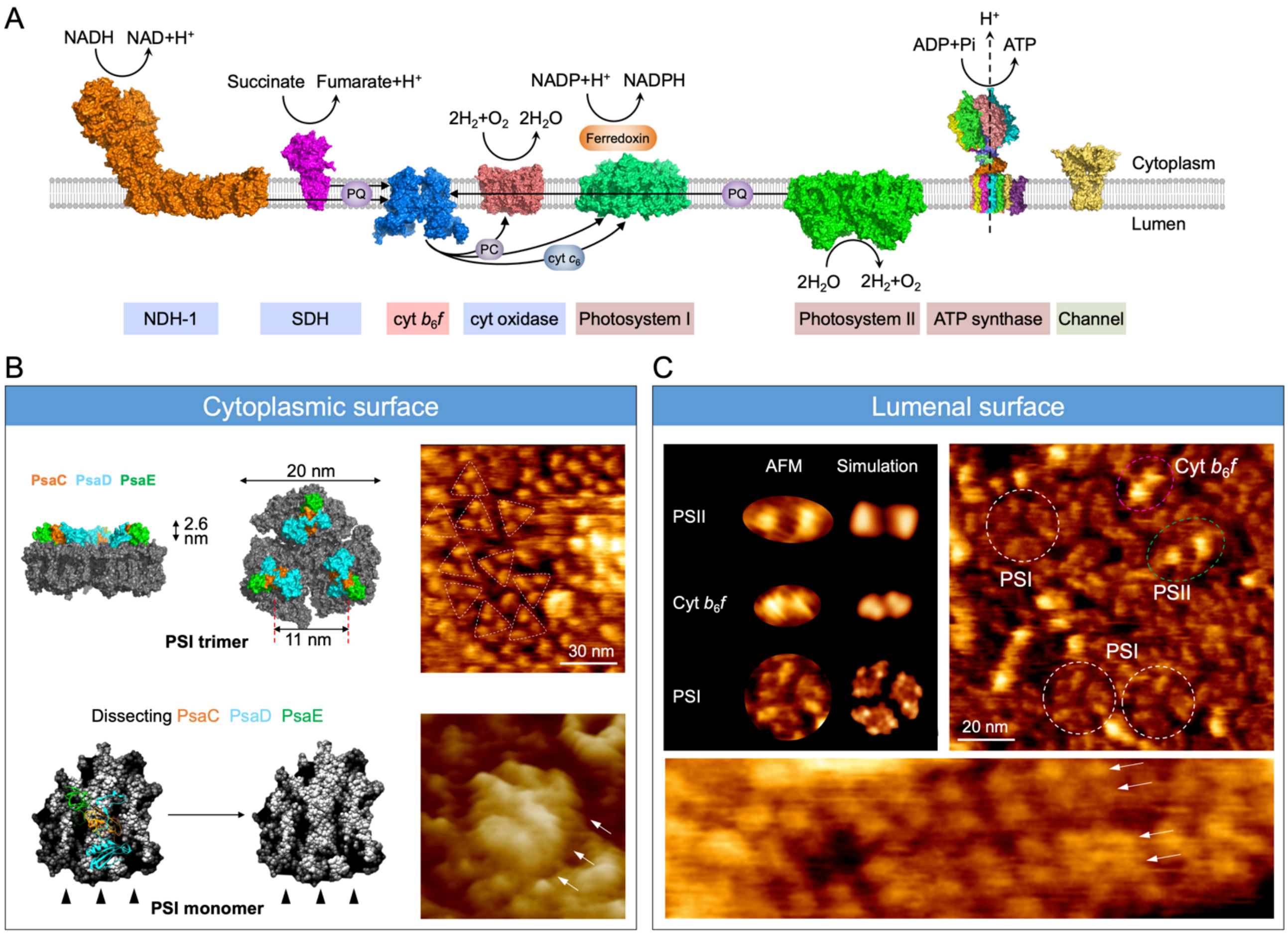

Supplemental Figure S2. Determination of the cytoplasmic and lumenal surfaces of cyanobacterial thylakoid membranes. A, Schematic model of cyanobacterial thylakoid membrane, adapted from (Liu, 2016). Photosystem I (PSI) represents the predominant complex in cyanobacterial thylakoid membranes and has a high protrusion on the cytoplasmic surface, whereas photosystem II (PSII) and cytochrome b_6f (Cyt b_6f) are poorly protruded from the same membrane surface but have greater protrusions on the lumenal surface than PSI. PC represents plastocyanin. PQ represents plastoquinone. **B**, Structural features of PSI trimers on the cytoplasmic surface of cyanobacterial thylakoid membrane. The notable features on the cytoplasmic side of cyanobacterial thylakoid membranes visualized by atomic force microscopy (AFM) are the protruding domains with a threefold rotational symmetry (upper right corner, triangles, the same image as shown in Figure 1C), formed by three extrinsic PSI subunits (PsaC, PsaD and PsaE), in agreement with previous AFM studies (MacGregor-Chatwin et al., 2019; Zhao et al., 2020). Furthermore, these protrusions could be removed by AFM nanodissection, displaying the detailed surface texture underneath indicated by arrows (bottom right corner) (Zhao et al., 2020). Another landmark feature at the cytoplasmic surface is the highly protruding domain of NDH-1 complex, as shown in Figure 1 and 5. **C**, Structural features of PSII dimers, Cyt b_6f dimers, and PSI trimers on the lumenal surface of cyanobacterial thylakoid membrane. Figures were adapted from (Zhao et al., 2020). The lumenal structure of PSI has less protrusion from the thylakoid surface and the lumenal surface of thylakoid membrane appears relative smoother under AFM compared to the cytoplasmic surface. PSII and Cyt b_6f protrusions were observed in the form of dimers at the lumenal side of thylakoid membrane, and thereby protrusions of dimeric arrays could be readily imaged in AFM imaging (indicated by arrows, see also Supplemental Figure S6). These detectable structural features allowed us to distinguish the cytoplasmic and lumenal surfaces of cyanobacterial thylakoid membranes.

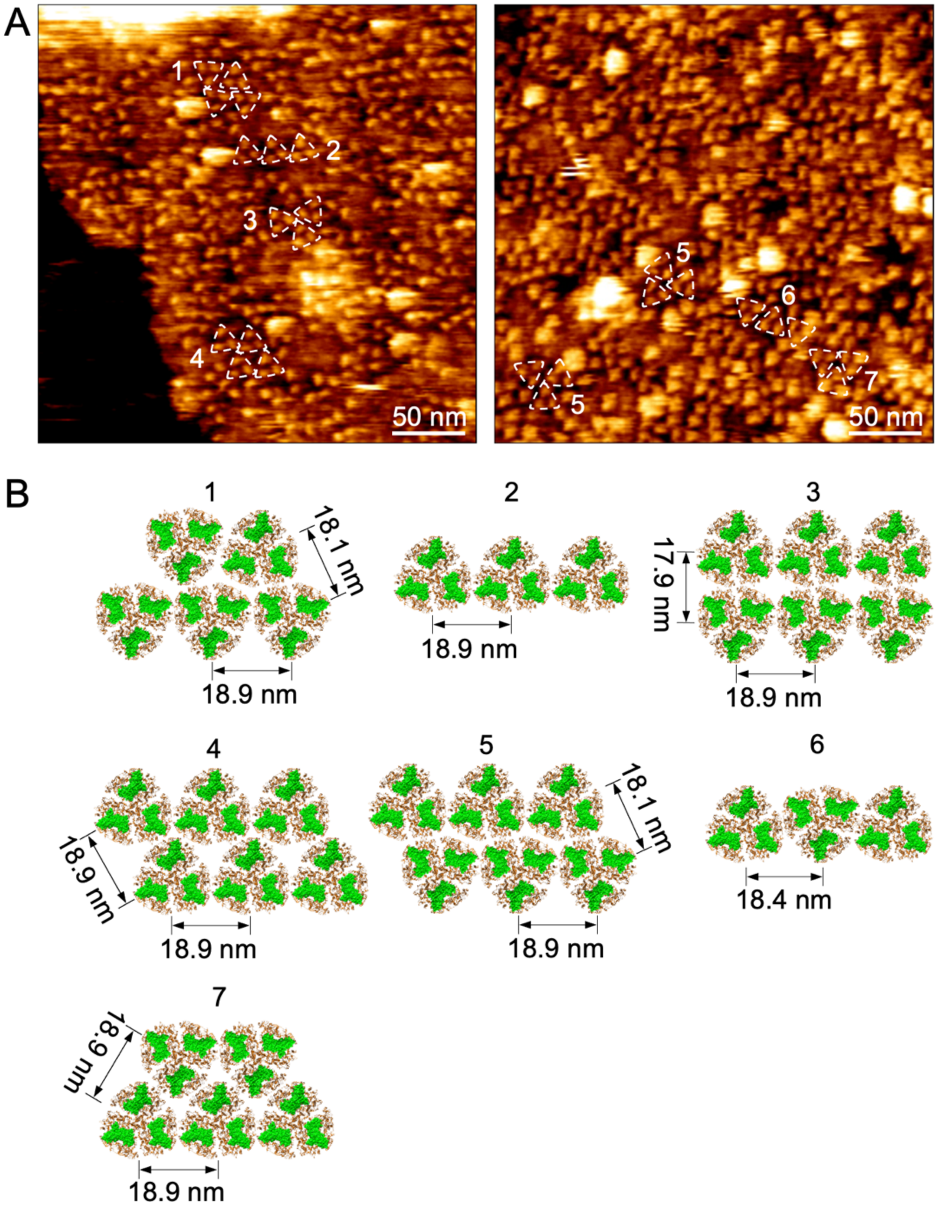

Supplemental Figure S3. Arrangement of photosystem I (PSI) trimers in thylakoid membranes from low light (LL)-adapted Syn2973 cells. A, High-resolution atomic force microscopy (AFM) images of the cytoplasmic surface of LL thylakoid membranes. The right panel is the zoom-in view of Figure 1A and shows the same membrane area as depicted in Figure 4F. Different types of PSI trimer oligomerizations (triangles) are indicated as 1, 2, 3, 4, 5, 6 and 7. **B**, Structural models of PSI trimer oligomerizations constructed using the PSI crystal structure (PDB: 1JB0) corresponding to the 7 arrangement patterns of PSI trimers observed in **A**. The spaces between adjacent PSI trimer were determined.

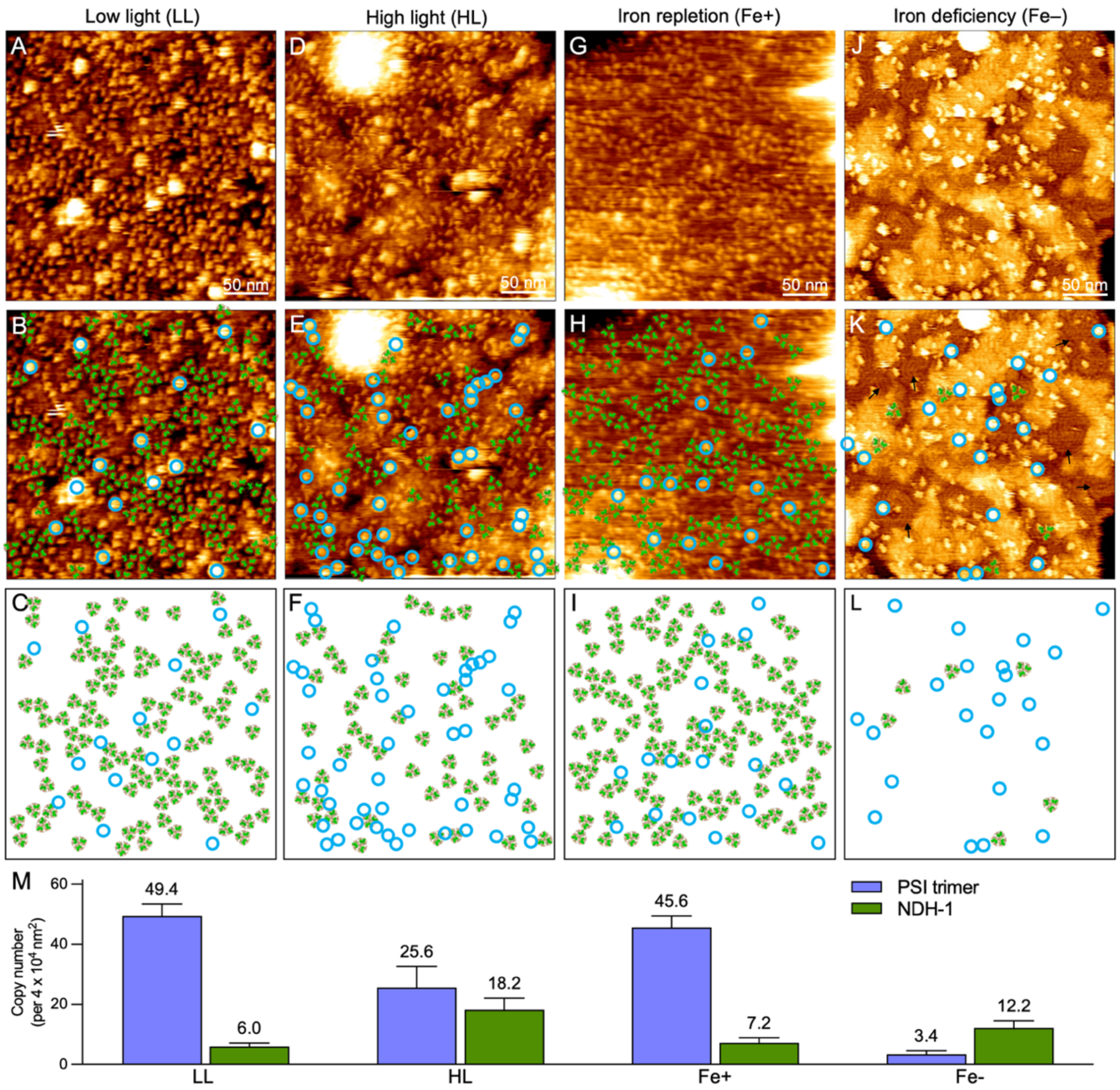

Supplemental Figure S4. The organization of photosystem I (PSI) trimers and putative NDH-1 in thylakoid membranes from low light (LL), high light (HL), Fe^+ and Fe^--adapted Syn2973 cells. A, Atomic force microscopy (AFM) image of the cytoplasmic surface of LL thylakoid membranes. This panel is the zoom-in view of Figure 1A and shows the same membrane area as depicted in Figure 4F and the right panel of Supplemental Figure S3A. **B**, The same image as **A** with PSI trimers and putative NDH-1 complexes highlighted by PSI crystal structure (green, PDB: 1JB0) and blue circle, respectively. **C**, Model of the arrangement of PSI trimers and putative NDH-1 complexes from **B**. **D**, AFM image of the cytoplasmic surface of HL thylakoid membranes. Zoom-in view of Figure 1E. **E**, The same image as **D** with PSI trimers and putative NDH-1 complexes highlighted by PSI crystal structure and blue circle, respectively. **F**, Model of the arrangement of PSI trimers and putative NDH-1 complexes from **E**. **G**, AFM image of the cytoplasmic surface of Fe+ thylakoid membranes. **H**, The same image as **G** with PSI trimers and putative NDH-1 complexes highlighted by PSI crystal structure and blue circles, respectively. **I**, Model of the arrangement of PSI trimers and putative NDH-1 complexes from **H**. **J**, AFM image of the cytoplasmic surface of Fe– thylakoid membranes. The same image was shown in Figure 5E. **K**, The same image as **J** with PSI trimers and putative NDH-1 complexes highlighted by PSI crystal structure and blue circle, respectively. Arrows indicates IsiA assemblies. **L**, Model of the arrangement of PSI trimers and putative NDH-1 complexes from **K**. **M**, The densities of PSI trimers and NDH-1 complexes in thylakoid membranes (per 4×10^4 nm^2) under LL, HL, Fe+, Fe– based on AFM topographs ($n = 5$). The copy number of PSI trimers per 4×10^4 nm^2 was 49.4 ± 4.0 under LL, 25.6 ± 7.0 under HL, 45.6 ± 3.8 under Fe+, 3.4 ± 1.2 under Fe–. The copy number of NDH-1 per 4×10^4 nm^2 was 6.0 ± 1.1 under LL, 18.2 ± 3.9 under HL, 7.2 ± 1.7 under Fe+, 12.2 ± 2.3 under Fe–. The representative AFM images were shown from at least five independent membrane preparations.

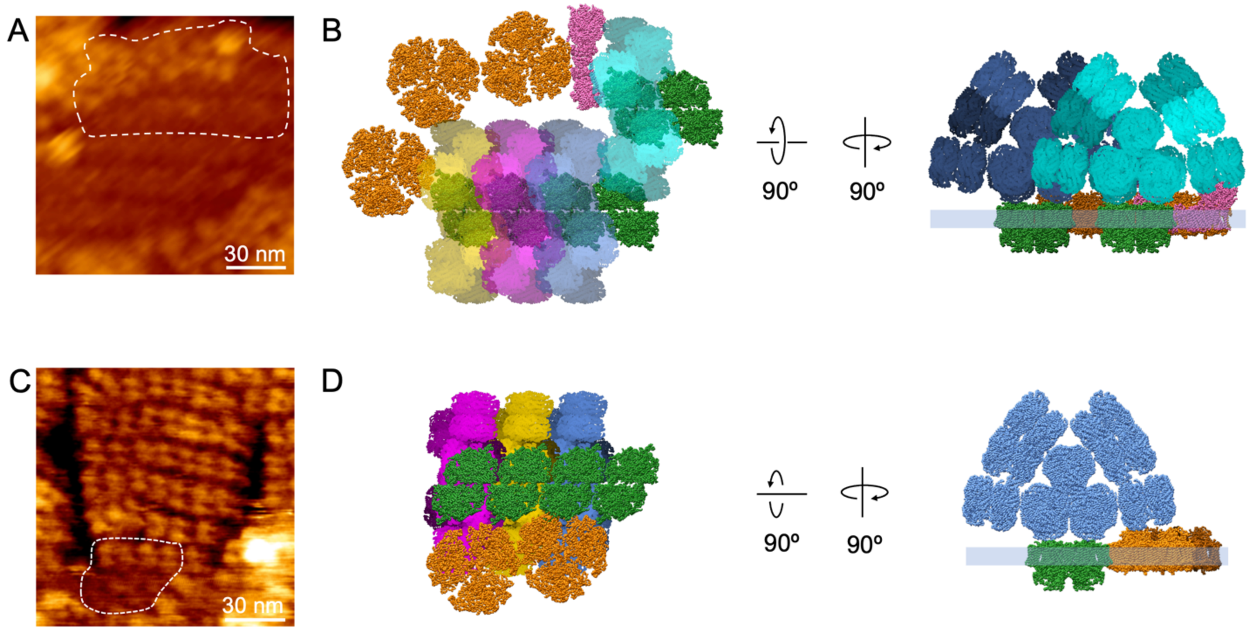

Supplemental Figure S5. Association of photosystem I (PSI) and photosystem II (PSII) dimer arrays in thylakoid membranes from low light (LL)-adapted Syn2973 cells. A, Atomic force microscopy (AFM) image of the thylakoid membrane cytoplasmic surface showing the arrangement of PSI and PSII dimer arrays. This panel is the zoom-in view of Figure 2B. **B**, Structural model of the arrangement of PSI (orange), NDH-1 (purple) with the arrays of PSII dimers (green) based on the AFM image shown in **A**. Phycobilisome structures are docked on top of the PSII arrays. **C**, AFM image of the thylakoid membrane lumenal surface showing the arrangement of PSI and PSII dimer arrays. This panel is the zoom-in view of Figure 3A, 3B, 3C, 3E. **D**, Structural model of the arrangements of PSI (orange) and the arrays of PSII dimers (green) based on the AFM image shown in **C**, with phycobilisomes docked on top of PSII. PDB ID: PSI (1JB0), PSII (3WU2), NDH-1 (6TJV), phycobilisome (7EXT).

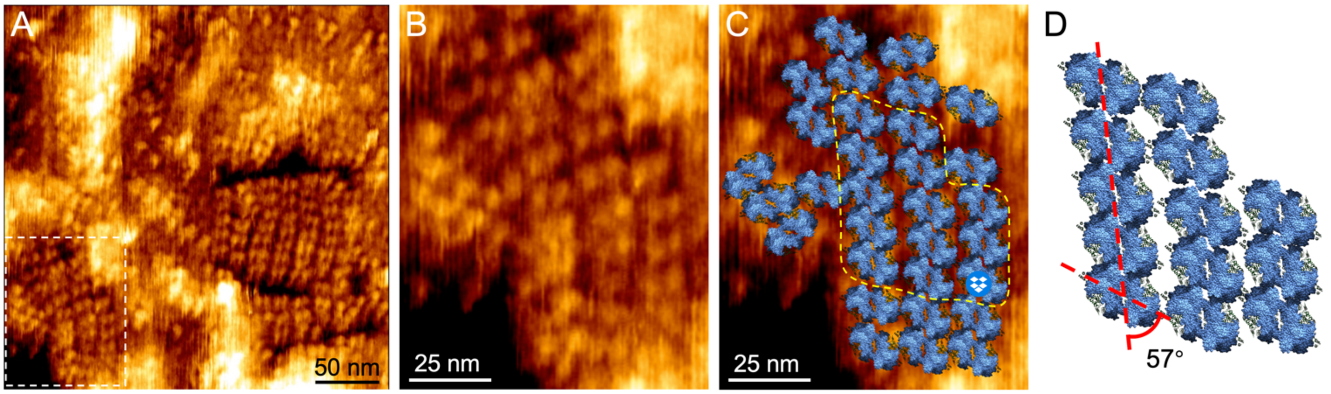

Supplemental Figure S6. Atomic force microscopy (AFM) images revealing the photosystem II (PSII) arrays on the lumenal surface of thylakoid membranes from low light (LL)-adapted Syn2973 cells. A, High-resolution AFM image of the lumenal surface of thylakoid membrane showing the ordered arrays of PSII dimers. This is the same image as shown in Figure 3B with a rotation angle of 90°. The area represented by white box is shown in **B**. **B**, Zoom-in view of the area highlighted in **A** showing the ordered arrays of PSII dimers. The same membrane area as shown in the bottom panel of Supplemental Figure S2C. **C**, Structural model of the arrangement of PSII dimers in ordered arrays in the thylakoid membrane (PDB: 3WU2). The area indicated by yellow dashed line is shown in **D**. **D**, Structural model of the arrangement of PSII arrays highlighted in **C**. The angle between the extension of PSII arrays and the direction of PSII dimer long axis is 57°.

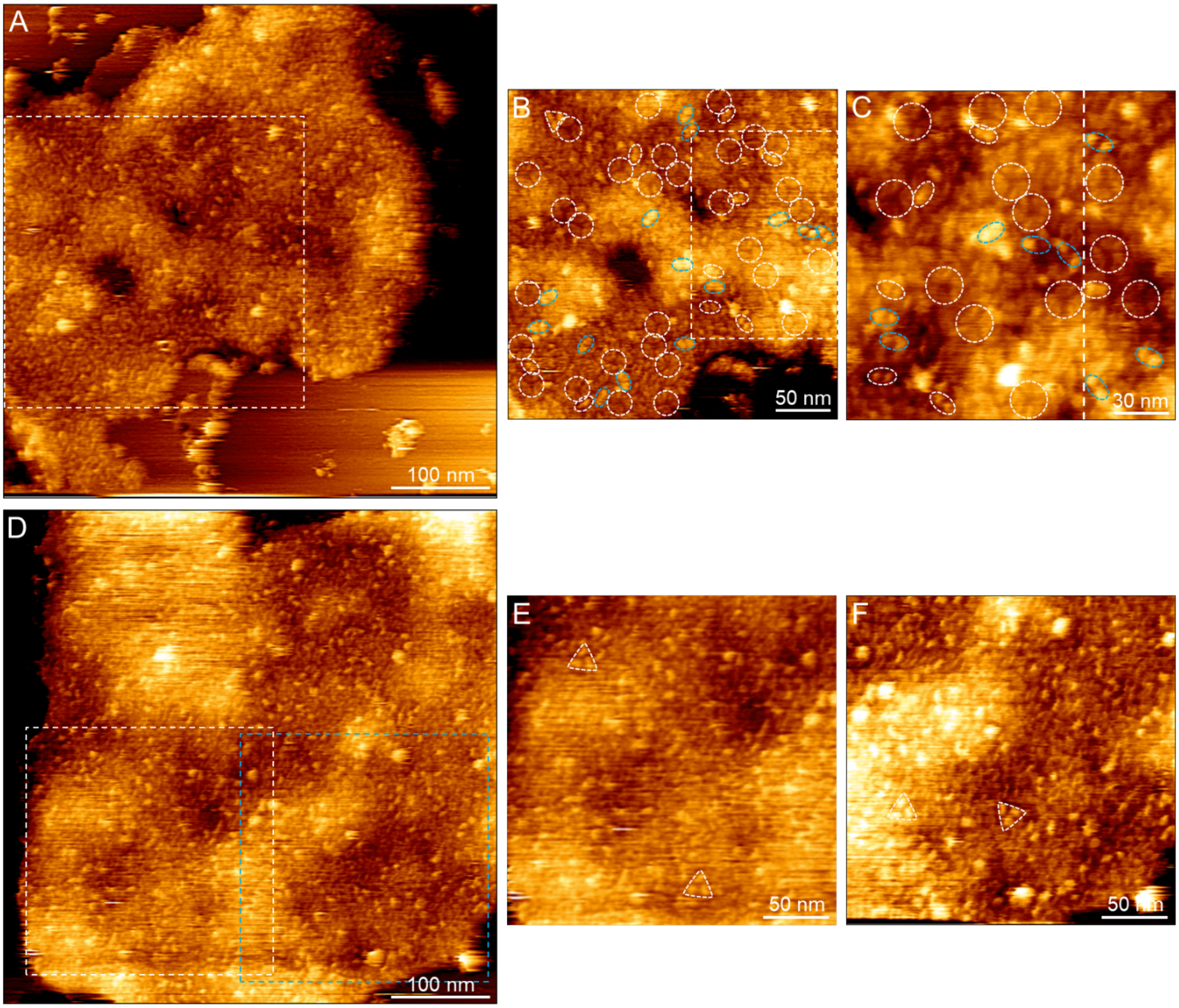

Supplemental Figure S7. Atomic force microscopy (AFM) image of the lumenal surface of thylakoid membrane from high light (HL)-adapted Syn2973 cells showing the distribution of photosynthetic membrane proteins. **A**, AFM overview image of the lumenal surface of thylakoid membrane fragment at a large scale. The area represented by white box is shown in **B**. **B**, Zoom-in view of the area highlighted in **A**. Photosystem I (PSI) trimers, photosystem II (PSII) dimers and cytochrome b_6f (Cyt b_6f) dimers highlighted by white circle, blue oval, and white oval, respectively. The area represented by white box is shown in **C**. **C**, Zoom-in view (the area at the left side of the white dashed line) of the area highlighted in **B**. **D**, Long-range AFM image of the lumenal surface of thylakoid membrane fragment. The area represented by white box and blue box is shown in **E** and **F**, respectively. **E-F**, Zoom-in view of the area highlighted in **D**. Trimeric PSI structures with the cytoplasmic side facing to the AFM probe in **B**, **E** and **F** are highlighted by triangles. The representative AFM images were shown from at least three independent membrane preparations.

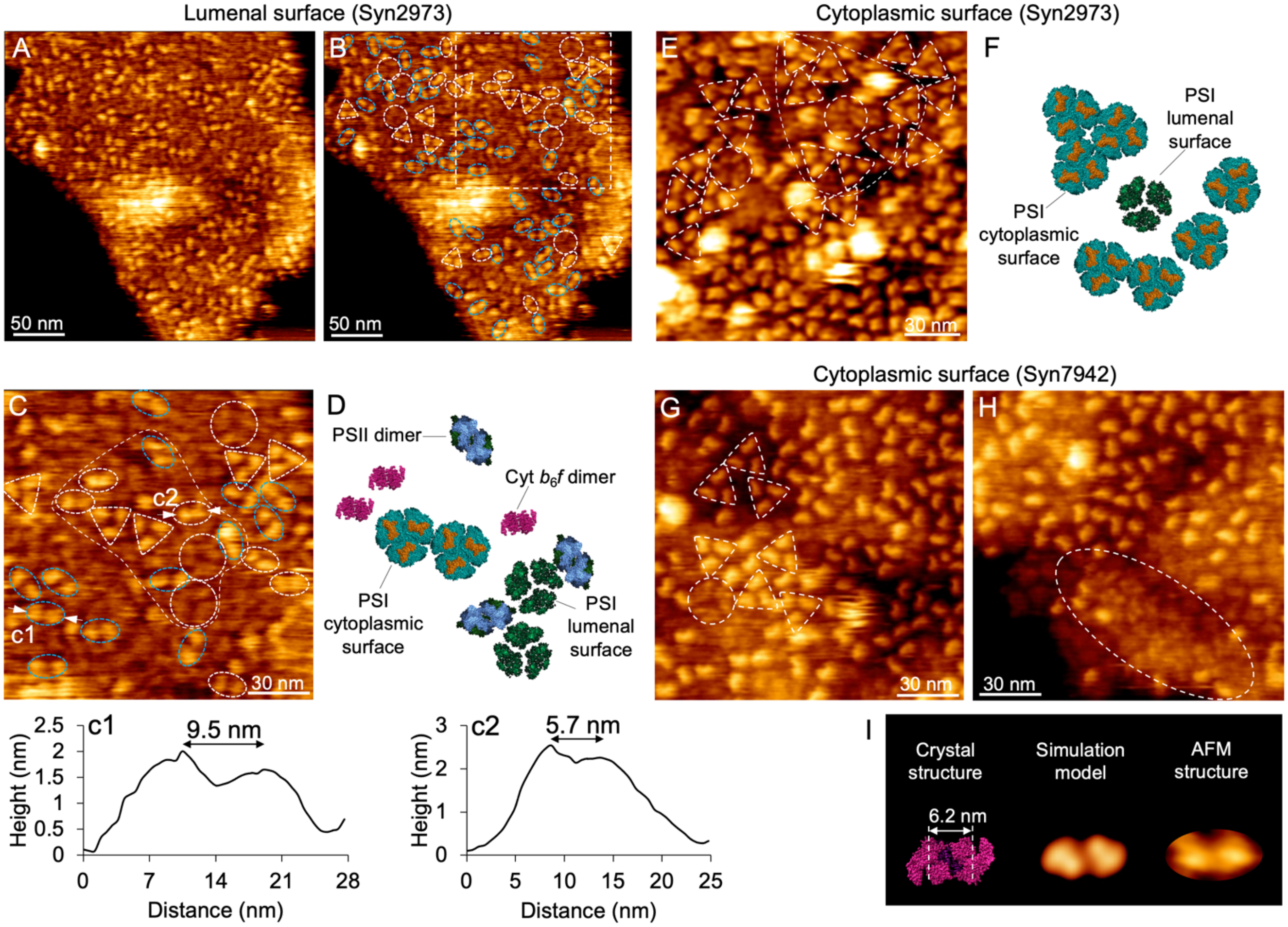

Supplemental Figure S8. Atomic force microscopy (AFM) images revealing the photosystem II (PSII), cytochrome *b₆f* (Cyt *b₆f*) and upside-down photosystem I (PSI) complexes in thylakoid membranes. A, AFM image of the lumenal surface of thylakoid membrane from low light (LL)-adapted Syn2973 cells. **B**, The same image as **A** with normal PSI trimers, PSII dimers and Cyt b_6f dimers highlighted by white circle, blue oval and white oval, respectively. Trimeric PSI structures with the cytoplasmic surface facing the AFM probe are highlighted by triangles. The area represented by white box is shown in **C**. **C**, Zoom-in view of the area highlighted in **B**. White arrows indicate the positions of height profiles. The lateral distance between peaks of PSII and Cyt b_6f is 9.9 ± 0.7 nm (n = 30) and 6.4 ± 1.2 nm (n = 12), respectively. **D**, Structural model of the arrangement of PSII dimer (blue), Cyt b_6f (purple), typical PSI (olive green), and “upside-down” PSI (orange-viridian) based on the AFM image in **C**. **E**, AFM of the cytoplasmic surface of thylakoid membrane from LL-adapted Syn2973 cells with normal PSI trimers highlighted by triangles. Trimeric PSI structures from the lumenal surface are highlighted by circles. This is the same image as shown in Figure 4G and zoom-in view of Figure 4F and the right panel of Supplemental Figure S3A. **F**, Structural model of the arrangement of typical PSI (orange-viridian) and “upside-down” PSI (olive green) based on the AFM image in **E**. **G**, AFM image of the cytoplasmic surface of thylakoid membrane from LL-adapted Syn7942 cells with normal PSI trimers highlighted by triangles. Trimeric PSI structures with the lumenal side facing the AFM probe are highlighted by circles. **H**, AFM image of the cytoplasmic surface of thylakoid membrane from LL-adapted Syn7942 cells. Membrane area occupied by PSI structures from the lumenal surface is highlighted by oval. **I**, Atomic structure, simulated AFM images based on PDB (middle) and AFM topograph (right) of Cyt b_6f from the lumenal surface. PDB ID: PSI (1JB0), PSII (3WU2), Cyt b_6f (2E74).

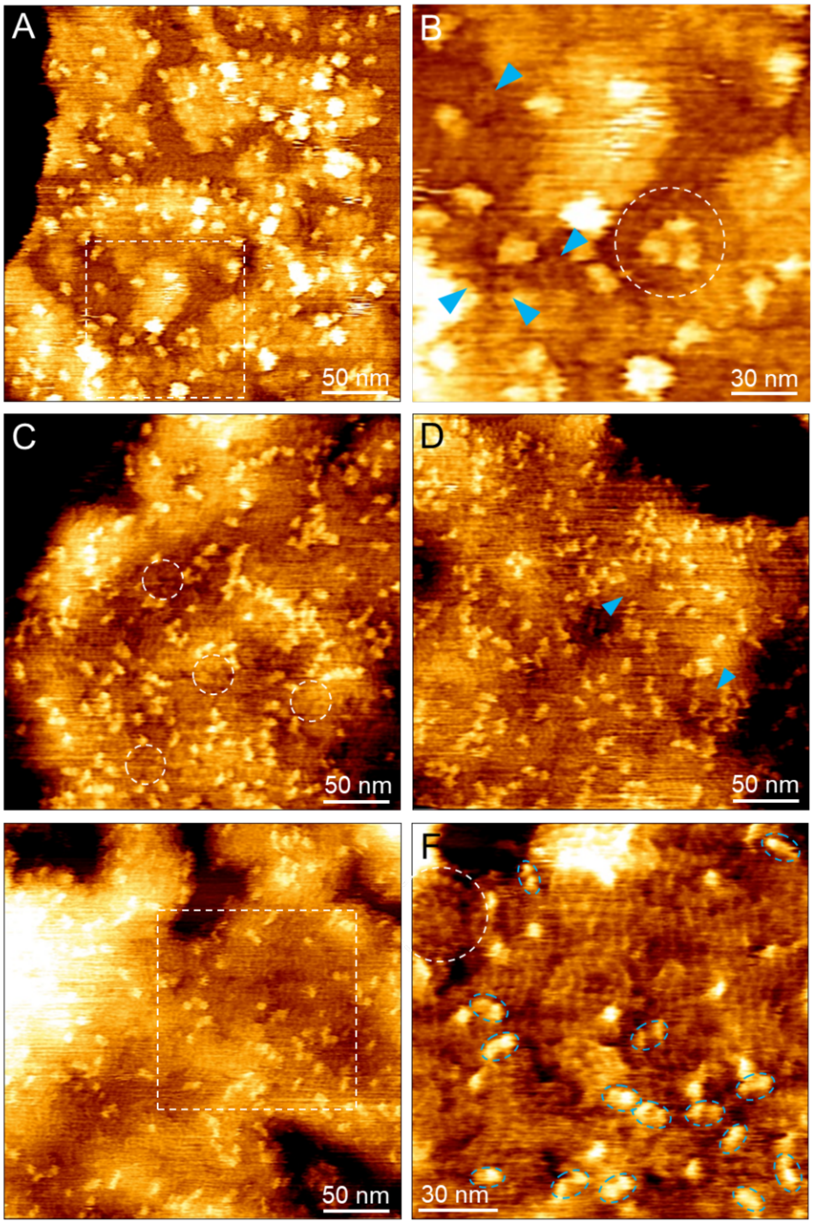

Supplemental Figure S9. Atomic force microscopy (AFM) images of thylakoid membranes from iron-starved (Fe–) Syn2973 cells. A, AFM topograph of the cytoplasmic surface of Fe– thylakoid membranes. The area delineated by the white box is shown in **B**. **B**, Zoom-in view of the area highlighted in **A** showing IsiA-PSI trimer (white circle) and putative ATPase rings indicated by blue arrows. **C**, AFM topograph of the lumenal surface of the Fe– thylakoid membrane fragment. The trimeric PSI are indicated by white circles. **D**, AFM topograph of the lumenal surface of the Fe– thylakoid membrane fragment with putative ATPase rings indicated by blue arrows. **E**, AFM topograph of the lumenal surface of the Fe– thylakoid membrane fragment. The area delineated by white box is shown in **F**. **F**, Zoom-in view of the area highlighted in **E** showing the IsiA-PSI trimer (white circle) and dimeric structures (blue oval). The representative AFM images were shown from at least five independent membrane preparations.

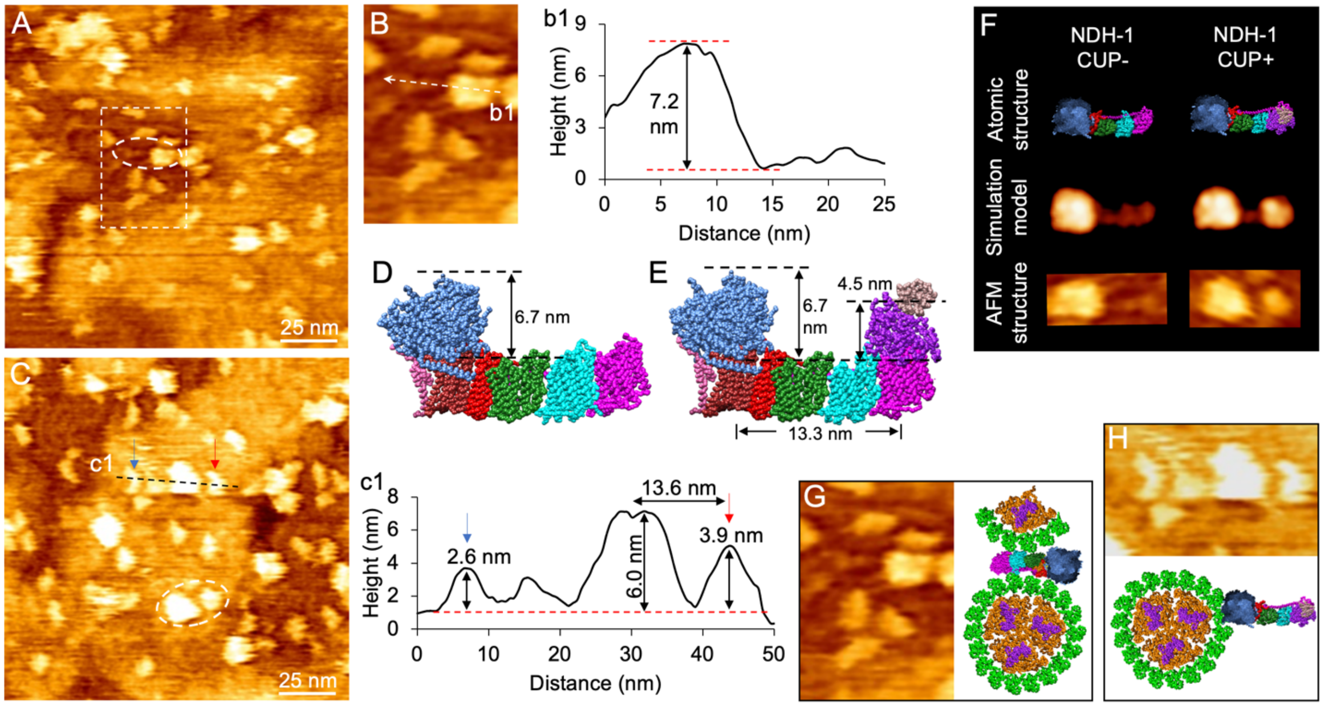

Supplemental Figure S10. Atomic force microscopy (AFM) images of putative NDH-1 in Fe– thylakoid membranes from Syn2973 cells. **A**, AFM image of the cytoplasmic surface of thylakoid membranes. The putative NDH-1 complex is highlighted with dashed oval. The area represented by white box is shown in **B**. **B**, Zoom-in view of the area highlighted in **A** showing the putative NDH-1 complex. Height profile b1 corresponds to the dashed line b1, with the height of the protruding domain above membrane surface shown. **C**, AFM image of the cytoplasmic surface of thylakoid membranes. It is the zoom-in view of Figure 5D, 5E, and Supplemental Figure S4J. The putative NDH-1 complex is highlighted with dashed oval. Height profile c1 corresponds to the dashed line c1, with lateral distances and heights of the protrusions above membrane surface shown. **D**, Front view of the atomic structure of NDH-1 without CUP (NDH-1 CUP-) (PDB: 6HUM) with the height of the hydrophobic arm shown. **E**, Front view of the atomic structure of NDH-1 with CUP (NDH-1 CUP+) (PDB: 6TJV) with the size shown. **F**, Atomic structure (top), simulated AFM images based on PDB (middle) and AFM topograph (bottom) of NDH-1 CUP- complex and NDH-1 CUP+ complex from the cytoplasmic surface. **G**, Structural model of the association of IsiA–PSI and NDH-1 complex (left) based on AFM topograph (right). **H**, Structural model of the association of IsiA–PSI and NDH-1 complex (bottom) based on AFM topograph (top). The representative AFM images were shown from at least five independent membrane preparations.

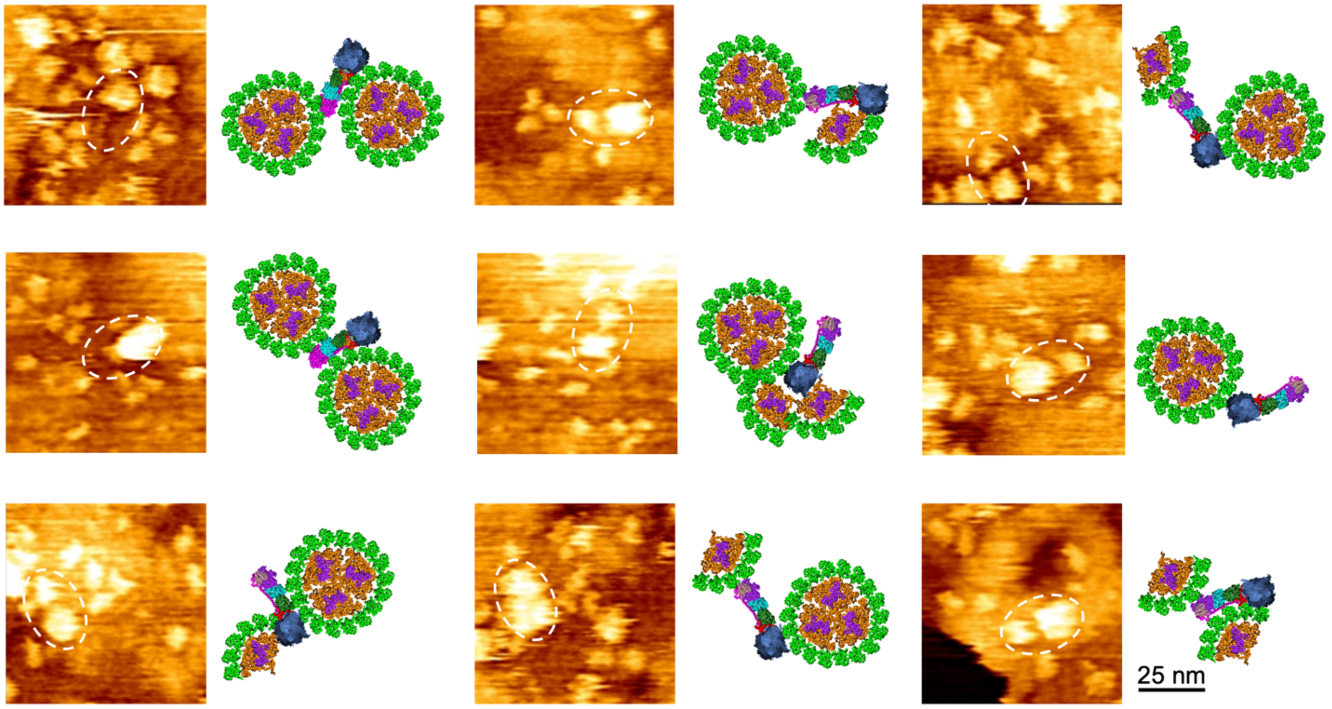

Supplemental Figure S11. Variability of the association of photosystem I (PSI) and NDH-1 in thylakoid membranes from iron-starved Syn2973 cells. Putative NDH-1 complexes are highlighted with dashed ovals. Models were constructed based on AFM topographs. The representative AFM images were shown from at least five independent membrane preparations.

Supplemental References

Liu LN (2016) Distribution and dynamics of electron transport complexes in cyanobacterial thylakoid membranes. Biochim Biophys Acta Bioenerg **1857:** 256-265

MacGregor-Chatwin C, Jackson PJ, Sener M, Chidgey JW, Hitchcock A, et al. (2019) Membrane organization of photosystem I complexes in the most abundant phototroph on Earth. Nat Plants **5:** 879-889

Zhao LS, Huokko T, Wilson S, Simpson DM, Wang Q, et al. (2020) Structural variability, coordination and adaptation of a native photosynthetic machinery. Nat Plants **6:** 869-882
